# Suppression of Sensorimotor Alpha Power Associated With Pain Expressed by an Avatar: A Preliminary EEG Study

**DOI:** 10.3389/fnhum.2018.00273

**Published:** 2018-07-09

**Authors:** Christian C. Joyal, Sarah-Michelle Neveu, Tarik Boukhalfi, Philip L. Jackson, Patrice Renaud

**Affiliations:** ^1^Laboratory of Virtual Reality Applications in Psychiatry (ARVIPL), Research Center, Philippe-Pinel Institute of Montreal, Montreal, QC, Canada; ^2^Cognition, Neuroscience, Affect and Behavior Research Group (CogNAC), Psychology Department, University of Quebec at Trois-Rivières, Trois-Rivières, QC, Canada; ^3^Psychology Department, University Mental Health Institute of Quebec (CRIUSMQ) and Laval University, Quebec, QC, Canada; ^4^Psychology Department, University of Quebec in Outaouais, Gatineau, QC, Canada

**Keywords:** empathy, assessment, electroencephalography, qEEG, perspective taking, alpha suppression, avatar

## Abstract

Several studies using functional magnetic resonance imaging (fMRI) showed that empathic capabilities are associated with the activation (and deactivation) of relatively specific neural circuits. A growing number of electroencephalography studies also suggest that it might be useful to assess empathy. The main goal of this study was to use quantitative electroencephalography (qEEG) to test whether observation of pain expressed by an avatar (virtual reality) induces a suppression of alpha waves over sensorimotor cortical areas, as it is observed with human stimuli. Not only was it the case, but also the magnitude of alpha suppression was correlated with perspective-taking capacity of participants. Both empathy levels and magnitude of sensorimotor alpha suppression (SAS) were significantly higher in women than men. Interestingly, a significant interaction emerged between levels of individual empathy and specificity of experimental instructions, where SAS in participants with good perspective-taking was higher during passive observation of the distressed avatar, while the opposite was true during an active (trying to understand) condition. These results suggest that: (1) synthetic characters are able to elicit SAS; (2) SAS is indeed associated with perspective-taking capacities; (3) Persons with poorer perspective-taking capacities can show significant SAS when proper instructions are provided. Therefore, qEEG represents a low-cost objective approach to measure perspective-taking abilities.

## Introduction

Currently, in clinical settings, empathy is usually assessed through self-report questionnaires (i.e., the Interpersonal Reactivity Index, Davis, 1983), which are highly susceptible to bias due to false responses, social desirability, and other factors (e.g., Van de Mortel, 2008). Identifying a neurobiological correlate of empathy would make it possible to develop an objective assessment. Several experimental studies have demonstrated that functional magnetic resonance imaging (fMRI) allows the visualization and quantification of key cortical and subcortical brain activation associated with empathy (e.g., Jackson et al., 2005; see Bernhardt and Singer, 2012; for a review). fMRI, however, is costly and not accessible in most clinical or forensic settings. The main goal of the present study was to confirm the capacity of a low-cost, available, portable neuroimaging technique—quantitative electroencephalography (qEEG)—to evaluate empathy levels. EEG records local electric potentials generated by the cerebral cortex and qEEG mathematically breaks down the frequency spectrum of electrical waves. This approach allows measurement of regional (different cortical areas) power (or amplitude) of particular oscillatory bands with high precision for both frequency (e.g., 1-Hz gradient) and time (1-s gradient). Given that empathic traits and states are associated with regional cortical modulation, qEEG might be useful to evaluate an individual’s empathy levels.

One of the most exciting discoveries in neuroscience over the past half-century is that of mirror neurons, i.e., fronto-parietal cortical cells that are activated (mirroring) in human and non-human primates during observation of a congener performing an act or behavior (Rizzolatti and Craighero, 2004). Mirror neuron responses activate the primary motor cortex (Hari et al., 1998; Muthukumaraswamy et al., 2004), forming a neural system that is hypothesized to promote prosocial behaviors through linking perception (seeing and understanding someone in distress) to action (providing assistance; Preston and de Waal, 2002; Pineda, 2005). Given that similar reactive activations are also observed in the somatosensory cortex (the other side of the central fissure) when a person observes painful stimuli or another person in pain (e.g., Avenanti et al., 2005; see Keysers and Gazzola, 2010; for a review), both primary motor and somatosensory cortices represent an important part of the cortical areas involved in the neural foundations of social interactions (Perry et al., 2011; Vanderwert et al., 2013) and empathy (Bernhardt and Singer, 2012).

qEEG is perfectly suited to measure sensorimotor cortical activation. First, sensorimotor cortex correspond to middle electrodes (T7-C3-Cz-C4-T8), which are less sensitive to eye blinks than anterior electrodes. Second, regional cortical asynchrony induces a power reduction in alpha wave frequencies (8–13 Hz), especially those surrounding 8–10 Hz (Pfurtscheller and Lopes da Silva, 1999; Goldman et al., 2002), which is easily measured with Fast Fourier Transformation (FFT) of the EEG signal. The sensorimotor alpha suppression (SAS) is thought to reflect a downstream cortical activation generated by the mirror neurons (Pineda, 2005). Interestingly, a growing number of qEEG studies suggest that empathic capacities are also associated with the magnitude of SAS (Cheng et al., 2008a; Yang et al., 2009; Perry et al., 2010; Woodruff et al., 2011; Moore et al., 2012; Hoenen et al., 2013, 2015; see also Babiloni et al., 2012; for more anterior regions of interest; Ono et al., 2009; for using the corollary enhancement of beta power in central locations).

Both qEEG source localization (Yang et al., 2009; Moore et al., 2012), and magneto-encephalography source modeling (Whitmarsh et al., 2011) have confirmed that SAS associated with empathic response is highest above the sensorimotor cortical cortex, more particularly on the posterior bank of the central sulcus (Cheng et al., 2008b). Interestingly, SAS may be significantly stronger in women than in men (Cheng et al., 2006, 2008a; Yang et al., 2009; see also Han et al., 2008; Schulte-Rüther et al., 2008), which would be compatible with the well-established fact that women, in general, possess higher empathy capacities and social sensitivity than men (e.g., Baron-Cohen et al., 2005; Toussaint and Webb, 2005). Gender difference in SAS is not systematically reported, however (Perry et al., 2011), and deserves further investigation.

SAS induced by a painful expression exhibited by another person is viewed as a gating mechanism that disinhibits sensory cortices, helping to understand the pain of others (Whitmarsh et al., 2011; Moore et al., 2012). Peng et al. (2015) suggest that central (or sensorimotor) alpha oscillatory modulation associated with pain perception is determined by sensori-discriminative, affective motivational and cognitive-modulative aspects of pain experience. Therefore, qEEG represents a promising, accessible, and affordable technique for evaluating empathy and its sub-components that avoids any self-report bias.

qEEG might also help enhance empathy through neurofeedback. Neurofeedback is an operant learning technique based on brain-computer interfaces. Although evidence-based data with qEEG neurofeedback generally concerns attention deficits and impulsivity (Arns et al., 2014), it has been used successfully to train other capacities, both for clinical (Simkin et al., 2014) and non-clinical (Gruzelier, 2014) purposes. Among these trainable capacities is empathy (Cavazza et al., 2014; Moll et al., 2014; Yao et al., 2016).

However, many questions need to be addressed before SAS can be used as a correlate of empathy, let alone a neurofeedback target. First, some studies (but not all, e.g., Perry et al., 2011; Woodruff et al., 2011; Hoenen et al., 2013) do not demonstrate its specificity as they fail to provide comparisons with alpha suppression that occurs simultaneously in other (non-central) cortical regions. The occipital cortices, most notably, should be considered because it is well known that alpha power is suppressed posteriorly by visual stimulation or increased attention load (Thut et al., 2006).

Second, the majority of existing studies elicited SAS through presentation of simple motor stimuli (e.g., observing or performing movements; Perry et al., 2011; Woodruff et al., 2011). Only a handful of qEEG investigations used presentation of stimuli related to empathy (e.g., signs of distress or pain), and these stimuli were typically limited to body parts, usually the hands (e.g., hands being pricked, cut, or crushed; Yang et al., 2009; Perry et al., 2010). One exception used video clips of human actors in painful (sad) situations (Hoenen et al., 2013), although the mode of expression was verbal (story telling), which is not well suited for neurofeedback training. Past studies also used brief stimulus presentations, commonly ranging from 1.7 s to 5 s for pictures (e.g., Yang et al., 2009; Perry et al., 2010; Moore et al., 2012; Hoenen et al., 2015), up to 80 s for video clips (Cheng et al., 2008a; Woodruff et al., 2011; Hoenen et al., 2013). It remains to be seen whether SAS can also be detected with longer (and noisier) stimulus presentations (e.g., 120 s), which is mandatory for neurofeedback training (Budzynski et al., 2009).

Third, although visual stimulation and virtual reality represent the best options for conducting neurofeedback studies, very few EEG studies used virtual agents expressing pain to generate brain response associated with empathy (Cavazza et al., 2014). While it is known that avatars can elicit empathic responses in humans (e.g., Paiva et al., 2005; Cheetham et al., 2009; Joyal et al., 2014), their capacity to elicit SAS associated with empathy remains to be demonstrated (de Borst and de Gelder, 2015). This point is crucial to the eventual development of neurofeedback training, which could involve, for instance, presenting avatars expressing different types and intensities of emotions in response to brain wave characteristics of the participant.

Finally, empathy is a multifaceted construct and its sub-components should be considered separately in EEG studies. The four-factor model of empathy proposed by Davis (1983) is still the best validated model to date (Chrysikou and Thompson, 2016). According to that model, empathy encompasses four different capacities: (1) perspective taking, or the tendency to spontaneously adopt the psychological point of view of others; (2) cognitive fantasizing, or the tendency to transpose ourselves imaginatively into fictional situations; (3) empathic concern, or the tendency to have feelings of sympathy or compassion oriented toward others; and (4) personal distress, or the tendency to have self-oriented feelings of personal anxiety or fear in response to seeing others in distress (Davis, 1983; Chrysikou and Thompson, 2016). To be clinically relevant, a biological correlate of empathy should be sensitive to perspective taking, as a defect in this capacity is a classic predictor of low communication skills, antisocial behaviors, and interpersonal violence (Chandler, 1973). To date, two studies have reported that perspective-taking capacities have a modulating effect on SAS (Perry et al., 2010; Hoenen et al., 2013), and a third reported a specific and significant correlation between SAS and perspective-taking capacities, although the study was based on motor (moving fingers) stimuli (Woodruff et al., 2011). It remains to be seen if the same association would be observed within an empathic context, especially if the character presented is synthetic.

The main goal of this preliminary investigation was to assess the capacity of avatars to elicit empathy-related SAS, while considering extraneous factors such as non-central alpha suppression and presentation of equivalent stimuli not expressing pain. Another objective of this study was to measure the strength of association between SAS and individual levels of empathy and its sub-components. We attempted to confirm the superiority of women vs. men in empathy-related SAS. Finally, this study aimed at providing some guidelines for future neurofeedback protocols. The following hypotheses were tested: H1: the magnitude of alpha wave suppression elicited by observation of expressions of pain will be significantly higher in central (sensorimotor) than occipital electrodes; H2: a dynamic avatar expressing pain will elicit SAS with a magnitude significantly higher than that evoked by the same avatar not expressing pain; H3: the magnitude of SAS elicited by observation of expressed pain will be significantly associated with the empathy capacities of participants; H4: women will show, on average, a significantly higher magnitude of SAS during observation of expressed pain than men.

## Materials and Methods

### Participants

Based on effect sizes obtained in previous studies (e.g., Hoenen et al., 2013), 24 right-handed adults were recruited from the general population through online classified advertising to participate in this study (mean age: 26.7 ± 4.6, 20–40). The sample consisted equally of men and women (*n* = 12), all screened for histories of neurological or psychiatric conditions through phone pre-screening and an on-site questionnaire. Participants had to refrain from drinking alcohol for 24 h prior to testing, and from drinking coffee (or any beverage containing caffeine) and smoking tobacco for 12 h prior to testing.

### Questionnaires

Levels of empathy were assessed individually with a validated French version (Gilet et al., 2013) of the International Reactivity Index (IRI), a self-report questionnaire that assesses four components of empathy: perspective taking (the tendency to spontaneously adopt the psychological point of view of others), fantasizing (the tendency to transpose ourselves imaginatively into fictional situations), empathic concern (having feelings of sympathy or compassion toward others), and personal distress (self-oriented feelings of personal anxiety or fear in response to seeing others in distress; Davis, 1983; Chrysikou and Thompson, 2016). In the French version, items are rated on a 7-point scale ranging from 1 (does not describe me well) to 7 (describes me very well), generating higher mean scores than the original 5-point scale (1983).

### Neurophysiological Material and Virtual Reality Environment

**Figure 1:**
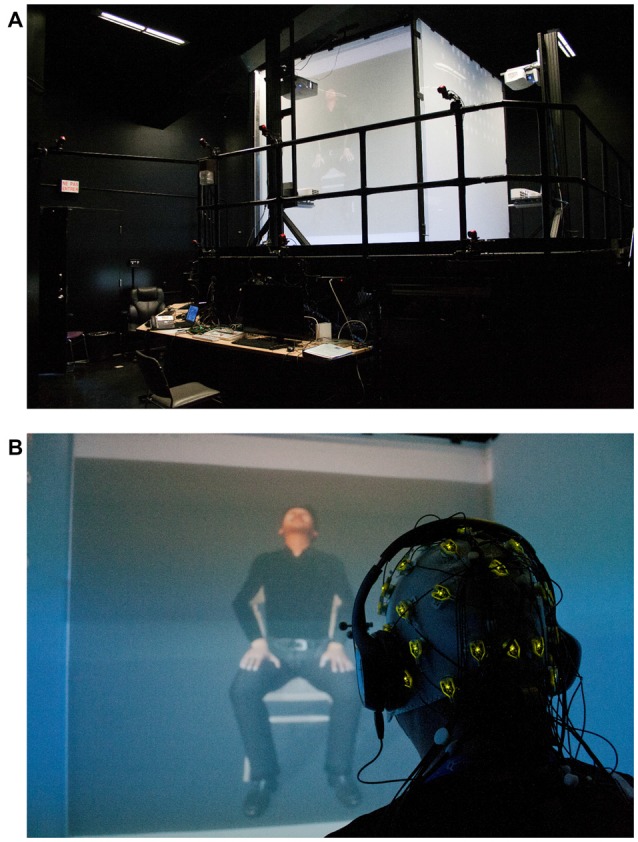
**(A)** The CAVE-like environment projecting the avatar; **(B)** a participant wearing the wireless EEG Acticap, headphones and the 3D goggles observing the avatar expressing pain.

A 32-electrode EEG cap (Acticap, extended 10–20 system, Brain Products, LLD) equipped with active wireless electrodes (ActiChamp amplifier paired with a MOVE system, Brain Products) was used to record electrical cortical activity (Figure 1). Participants were seated in a CAVE-like, 4-wall immersive environment (iCube, Viz-Tek Inc.) wearing 3D active shutter glasses (EdgeVR, Volfoni, Inc.) and headphones fitted with the wireless Acticap (Figure 1). This EEG system was chosen because it is particularly robust against electromagnetic fields (active electrodes with a co-integrated amplifier and noise subtraction; Metting Van Rijn et al., 1996; Usakli, 2010).

A 2-min period of habituation was first allowed, in which participants could freely explore the content of a neutral animation presented in 3D. Because simple observation of movement is known to suppress central alpha wave (e.g., Muthukumaraswamy et al., 2004), experimental conditions involved a non-moving avatar, a moving avatar, and the same moving avatar expressing pain. Stereoscopic stimuli were presented for 2 min each and consisted of the four following items (see Boukhalfi et al., 2015 for technical details): (1) change of the environment background from black to lighter gray (baseline condition, eye-open, luminosity modification); (2) presentation of a whole-body, adult Caucasian male avatar, seated in front of the same gray background, dressed in black and gray, audibly and regularly breathing (neutral condition); (3) presentation of the same seated avatar with the same audible breathing but moving forward and backward with hands on knees (movement condition); and (4) presentation of the same seated and moving avatar expressing pain facially and orally (pain condition). Facial expressions of emotions by these stimuli had been previously validated with another non-clinical sample of participants (Joyal et al., 2014).

Participants were asked either to simply observe the virtual character as an external object, without trying to understand or feel his emotions (Observation) or to try to understand and feel the emotion expressed by the virtual character (Empathy). Stimuli were presented six times in total—two different instructions (observe or empathize) across four different conditions (baseline, neutral, movement and expressed pain)—with the order of presentation counterbalanced for instruction (observation or empathy) and conditions (Neutral → Movement → Pain, or Pain → Movement → Neutral). Each presentation was followed by a 2-min rest period.

### Data Acquisition

This study is based on continuous qEEG signals recorded at bilateral sensorimotor (T7-C3-Cz-C2-T8) and occipital (O3-Oz-O2) sites (Recorder software, Brain Products). The more lateral T7 and T8 electrodes were added to the classical central electrodes (C3-Cz-C4) to include lateral parts of the sensorimotor cortex (Fabbri-Destro and Rizzolatti, 2008), usually overlooked by previous EEG studies. Data were referenced at FPz and re-referenced offline with bilateral mastoids to reduce eye-blink contamination. Electrical impedance was held below 25 kΩ, as required with active EEG electrodes. Data were sampled at 500 Hz and filtered with bandpass set at 0.01 Hz (high-pass) and 70 Hz (low-pass), and a notch filter for the 60 Hz frequency (North American electrical noise). Signals exceeding ± 100 μV (muscle artifacts) were automatically rejected.

### Data Analyses

Data processing followed the procedure of previous studies that used SAS to evaluate social cognition and empathy (Perry et al., 2011; Woodruff et al., 2011). First, both sensorimotor and occipital sites were recorded to make it possible to distinguish between alpha modulation generated by observation of expressed pain (sensorimotor) vs. alpha wave reaction to more general modifications of the visual environment (occipital) or attentional processes (widespread). Second, individual EEG outputs were inspected for artifacts by two independent raters (including a certified medical EEG technician) until a 100% inter-rater agreement was reached. At least 60 s of artifact-free EEG data was required (and obtained in all cases) to achieve minimal stability for spectral decomposition (qEEG). Third, records were divided into 1 s segments (minimum of 60 segments), and for each segment the integrated power in the 8–13 Hz range was computed using a Fast Fourier Transform (FFT) obtained at 1 Hz intervals with a Hanning window. Fourth, segments were averaged for each condition (baseline, neutral, movement and pain). Given that the absolute signal power is sensitive to individual differences (e.g., skull density, scalp thickness, baldness, electrode impedance), ratios were computed for each condition based on the baseline data (i.e., neutral/baseline; movement/baseline; pain/baseline). Finally, ratios were log transformed because they are not normally distributed (Pineda and Oberman, 2006). A negative log ratio under indicates a suppression of power compared to the baseline. Therefore, the dependant variable was a suppression index, calculated as the logarithm of the ratio of the alpha power during each condition relative to the alpha power during the baseline condition (Perry et al., 2011). Given our objective to obtain guidelines for neurofeedback protocols, exploratory analyses were conducted with 8–10 Hz vs. 8–13 Hz bands at each electrode (data not shown). In accord with early studies of SAS (Cochin et al., 1998, 1999), empathy-related SAS was optimal in the 8–10 Hz band. It was also highest, on average, at the T7 electrode (left lateral sensorimotor cortex). Therefore, all remaining analyses are based on 8–10 Hz suppression at T7.

### Statistical Analyses

An ANCOVA was conducted to control for occipital alpha suppression (covariate) and to test inter-factor interactions: 2 (gender) × 2 (level of dispositional empathy: low vs. high) × 2 (instruction: simply observe vs. empathize) with SAS as the dependant, repeated measure (neutral, movement, pain). Greenhouse-Geisser corrections were applied to the degree of freedom when necessary (i.e., if data sphericity was not attained). Effect sizes (corresponding to Pearson’s coefficient *r*) of 0.10, 0.30 and 0.50 were considered as small, medium and large, respectively. The strength of association between the magnitude of T7 alpha suppression and that of subcomponents of empathy was also assessed with *r* (bivariate Pearson comparisons).

### Ethical Considerations

A compensation of $50 was given to each participant at the end of the experiment. The study was approved by the ethics committees of the Philippe-Pinel Institute of Montreal and the University du Québec en Outaouais (UQO). This study was carried out in accordance with the recommendations of the Canadian “Tri-Council Policy Statement: Ethical Conduct for Research Involving Humans (TCPS)” with written informed consent from all subjects. All subjects gave written informed consent in accordance with the Declaration of Helsinki.

## Results

**Figure 2:**
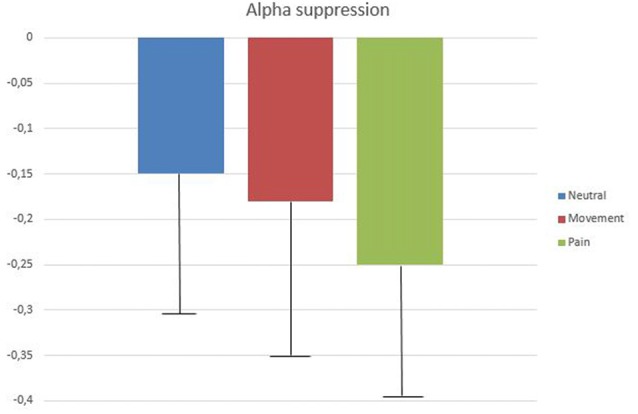
Mean log ratios (with standard deviation bars) of low alpha (8–10 Hz) amplitude difference between baseline (eye open, blue screen) and the three experimental conditions (neutral, movement and pain) at T7 (observation and empathic instructions).

The 2 (gender) × 2 (level of empathy) × 2 (instruction) ANCOVA (controlling for alpha modulation at occipital sites) with repeated measures (neutral, movement, and pain) confirmed that the three types of avatar presentation induced significant SAS (compared with the baseline condition), which was significantly greater during the pain condition (*M* = −0.25 ± 0.17) than during both the neutral (*M* = −0.146 ± 0.22) and movement (*M* = −0.18 ± 0.18) conditions (*F*_(1,39)_ = 3.83, *p* < 0.05; *r* = 0.49; *post hoc*: pain vs. neutral, *p* < 0.001; pain vs. movement, *p* = 0.003; neutral vs. movement, *p* > 0.05; Figure 2). Main effects for level of empathy (low: *M* = −0.216 ± 0.21 vs. high: *M* = −0.284 ± 0.25; *F*_(1,39)_ = 9.9, *p* = 0.03; *r* = 0.44) and gender (men: −0.229 ± 0.24 vs. women: −0.272 ± 0.23; *F*_(1,39)_ = 4.6, *p* < 0.05; *r* = 0.33) were also significant, with significantly higher alpha suppression in participants with high empathy scores and in women.

The effect of instructions (observe vs. empathize) was not statistically significant (*F*_(1,39)_ = 0.39, *p* > 0.05; *r* = 0.1). In fact, the simple observation of the avatar tended to induce greater SAS (all types of presentations still taken together), than trying to empathize with it (observe: *M* = −0.219 ± 0.23 vs. empathize: *M* = −0.166 ± 0.22; *F*_(1,47)_ = 2.69, *p* = 0.10, *r* = 0.22). No interaction was statistically significant.

Limiting the analyses to the pain condition revealed that the magnitude of SAS in both men (*M* = −0.377 ± 0.23) and women (*M* = −0.404 ± 0.24) with high empathy levels was significantly higher than that for both men (*M* = −0.206 ± 0.18) and women (*M* = −0.212 ± 0.21) with low empathy levels (*F*_(1,23)_ = 4.34, *p* = 0.05, *r* = 0.42). However, the interaction between the instruction condition (simply observing distress vs. attempting to empathize with the distressed avatar) and the level of empathy (low vs. high) approached significance (*F*_(1,47)_ = 2.96, *p* = 0.09, *r* = 0.27), indicating that simple observation tended to be sufficient to elicit significant central alpha suppression in persons with high levels of empathy, whereas attempting empathize with the avatar was necessary (and successful) to elicit alpha suppression in persons with low levels of empathy.

Finally, bivariate two-way Pearson correlations showed that the magnitude of SAS provoked by the distressed avatar was associated (nearly significant) with the perspective-taking capacities of the participants under the observing (*r* = 0.398, *p* = 0.054) but not the empathizing conditions. No other correlations approached significance.

## Discussion

The main goal of this study was to determine whether an avatar expressing pain can induce a significant SAS in humans. In accord with fMRI investigations (e.g., Gobbini et al., 2011), this study found that a synthetic agent can activate brain regions associated with empathy. It was found that SAS was evoked by observation of an avatar expressing pain and was significantly stronger than that evoked by the same avatar executing the same movement without expressing pain. Therefore, SAS was not simply due to viewing movements (e.g., Muthukumaraswamy et al., 2004). In addition, SAS did not simply reflect a posterior (occipital) alpha modulation associated with attentional processing (e.g., Thut et al., 2006), because the latter was entered as a co-variable in the analyses. These results represent a partial answer to the questions raised by de Borst and de Gelder (2015), who correctly wondered if using avatars for the neurobiological assessment of empathy is appropriate.

Another goal of this study was to confirm that SAS is associated with individual trait empathy or its sub-components. In accordance with Woodruff et al. (2011), it was found that the magnitude of SAS evoked by the observation of pain correlated with perspective-taking capacities, but not with the three other components. Therefore, the usefulness of SAS to assess empathy seems to concern perspective-taking capacities in particular.

As expected (Cheng et al., 2006, 2008a; Yang et al., 2009), a higher magnitude of SAS was found in women, on average, than in men. Also in accord with previous investigation (Moore et al., 2012), instructing participants to simply observe vs. to attempt to understand what the avatar was feeling had no effect, on average, on the magnitude of SAS. Interestingly, however, participants with higher empathy only needed to observe the distressed avatar to show significant SAS, whereas participants with lower empathy capacities needed to consciously try to understand the feelings of the avatar to show a significant SAS. These results suggest not only the presence of an automatic brain mechanism in empathic persons but also that the mechanism can be elicited through conscious effort. This possibility should be tested with clinical populations that lack empathy.

This study should not serve as a blueprint for neurofeedback paradigms, however. Although empathy-related SAS was higher, on average, at T7, it was not necessarily the case for all participants. As shown in the results, standard deviations (inter-individual variation) were elevated (much more than variations of empathy levels). Therefore, neurofeedback paradigms aiming at modulating empathy-related SAS will have to target cortical sites determined individually, as they might differ from one person to another.

In conclusion, this study confirms suggestions that qEEG and sensorimotor alpha waves can serve to assess empathy (Yang et al., 2009; Perry et al., 2010; Woodruff et al., 2011; Hoenen et al., 2013), and, more specifically, perspective-taking capacities. The effectiveness of using avatars to evaluate and, eventually, to train cerebral response associated with empathy was also demonstrated. The search for a single brain site to use in such training proved to be futile, however, as inter-subject variability was too high. While evaluative qEEG studies for empathy can make use of the average signals of all central electrodes, future neurofeedback protocols will have to determine individually which region is most effective for each participant. Finally, the usefulness of the present results should be confirmed in future qEEG studies that use participants with known empathy deficits recruited from clinical and forensic settings.

## Author Contributions

CJ initiated and contributed to experimental design, conducted the literature review, analyzed and interpreted the data and wrote the manuscript. S-MN collected the data and contributed to experimental design and data interpretation. TB created the stimuli, programed the presentation scripts and coordinated the multiplateform interface. PJ contributed to experimental design, data interpretation and revision of the manuscript. PR initiated and contributed to experimental design, revision of the manuscript and data interpretation.

## Conflict of Interest Statement

The authors declare that the research was conducted in the absence of any commercial or financial relationships that could be construed as a potential conflict of interest.
